# Commentary: Left ventricular assist device infections and epidemiologic literature—still more work to be done

**DOI:** 10.1016/j.xjon.2021.09.039

**Published:** 2021-09-29

**Authors:** J. Nathan Mynard, Massimo Baudo, Mohamed Rahouma

**Affiliations:** a Department of Cardiothoracic Surgery, Weill Cornell Medicine, New York, NY; b Department of Cardiac Surgery, Spedali Civili di Brescia, Brescia, Italy; c Department of Surgical Oncology, National Cancer Institute, Cairo, Egypt

**Figure:**
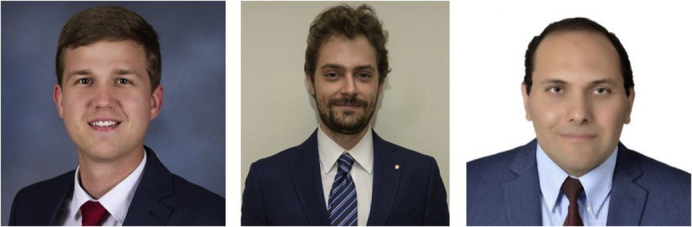
J. Nathan Mynard, MD, Massimo Baudo, MD, and Mohamed Rahouma, MD

**Central Message**

Left ventricular assist device infections are common, with driveline infections predominating. Further work is needed to reduce infection incidence and improve standardized epidemiologic reporting.

See Article page 405.

Pienta and colleagues^1^ present a thorough scoping review that effectively synthesizes the available literature regarding left ventricular assist device (LVAD) infections. As we continue to navigate the changing landscape of mechanical circulatory support technology, infections historically are the most common adverse event following LVAD implantation, with 41% of patients having a major infection at 1 year.^2^ This scoping review highlights that LVAD-associated infections remain a problematic obstacle to the care of patients with end-stage heart failure that previous works suggests portend an increased risk of additional morbidity, mortality, and health care costs (with a median of $11,506).^2^, ^3^, ^4^ Although high-quality data are lacking, the authors were able to present practical data that can guide clinicians and stimulate future research.

There are numerous ways to collate data to strengthen an evaluation of discrete clinical questions. At the top of the analytic pyramid are meta-analyses (MTAs), which occur in 3 main forms: single arm, pairwise, and network.^5^ In single-arm MTA, data are pooled in a quantitative manner from (ideally) high-quality randomized controlled trials that evaluate a single intervention with the goal of producing a single aggregate quantitative conclusion.^6^ Pairwise MTAs evaluate the pooled effects of 2 interventions, whereas network MTAs evaluate multiple interventions regardless of if direct comparisons are available.^6^, ^7^, ^8^ In contrast to the meta-analysis approach of statistically synthesizing the results of quantitative studies to answer a clinical question, a systematic review typically is composed of a more narrative approach.^5^ Finally, the major defining aspect of a scoping review like the current text is the lack of a truly defined final answer. A scoping review is often a great way to present data that are heterogenous and observational in nature.^5^ It can be thought of as a precursor or preliminary systematic review without the required quality assessment, thus functioning to synthesize available data and help guide future research.^5^ All of these methods derive their importance from transparent methodology that should be explicitly defined and reproducible by external researchers.

Finally, in addition to the relevant clinical takeaways from this review, an important topic of consideration the authors aptly pointed out is the lack of standardized reporting despite available guidelines. In this scoping review, Pienta and colleagues^1^ include 132 texts from 72 unique patient cohorts and do a great job in presenting their analysis in line with reporting guidelines from the 2011 International Society for Heart and Lung Transplantation (ISHLT) by grouping LVAD infections into 3 predefined categories: LVAD-specific, LVAD-related, and non–LVAD-related infections.^9^ Although use is increasing, only 36% (48/132) of the included manuscripts reported infection definitions in line with the ISHLT guidelines.^1^^,^^9^ In addition, few of the studies included in their analysis reported in conjunction with Strengthening the Reporting of Observational Studies in Epidemiology (STROBE) guidelines, including lacking significant portions of demographic and follow-up information.^1^^,^^10^ While some may argue that this may decrease the generalizability of this scoping review, it should be noted that the authors found the variance in infection rates persisted in groups adhering to the ISHLT guidelines.^1^ Acknowledging the poor adherence to reporting guidelines should serve to promote ongoing efforts to improve observational study reporting, especially as it pertains to LVAD-associated infections. Additional consensus statements have called for further standardized LVAD infection reporting in the hopes to improve research and treatment.^11^ The authors not only effectively present the available literature on an important clinical topic, but they have also highlighted needed quality improvement. The future is bright, but work is still to be done.
